# Headache among children and adolescents in Zambia: revised prevalence estimates, and estimates of attributed burden, from a national schools-based study

**DOI:** 10.1186/s10194-026-02438-4

**Published:** 2026-06-25

**Authors:** Nfwama Kawatu, Somwe Wa Somwe, Ornella Ciccone, Derya Uluduz, Tayyar Şaşmaz, Bengü Nehir Buğdaycı Yalçın, Christian Wöber, Andreas Kattem Husøy, Timothy J. Steiner

**Affiliations:** 1 Department of Paediatrics and Child Health, School of Medicine, University of Zambia, Lusaka, Zambia; 2 Neurology Department, Cerrahpaşa School of Medicine, Istanbul University, Istanbul, Turkey; 3 Public Health Department, School of Medicine, Mersin University, Mersin, Turkey; 4 Department of Neurology, Medical University of Vienna, Vienna, Austria; 5 NorHead, Department of Neuromedicine and Movement Science, Norwegian University of Science and Technology, Edvard Griegs gate, Trondheim, Norway; 6 Department of Neurology and Clinical Neurophysiology, St Olavs University Hospital, Trondheim, Norway; 7 Department of Neurology, University of Copenhagen, Copenhagen, Denmark; 8 Department of Brain Sciences, Imperial College London, London, UK

**Keywords:** Child and adolescent headache, Migraine, Tension-type headache, Medication-overuse headache, Undifferentiated headache, Diagnostic criteria, International Classification of Headache Disorders, Epidemiology, Burden of disease, Schools-based study, sub-Saharan Africa, Zambia, Global Campaign against Headache

## Abstract

**Background:**

In our earlier report of headache prevalence among children (6–11 years) and adolescents (12–17) in Zambia, we defined undifferentiated headache (UdH) conventionally as mild headache lasting < 1 h. However, we recognised diagnostic uncertainties, which also occurred in our similar studies elsewhere in sub-Saharan Africa (Ethiopia and Benin). Here we use a modified definition of UdH, making new estimates of prevalence accordingly, and estimates based on these of headache-attributed burden. The study was part of the global schools-based programme conducted by the Global Campaign against Headache.

**Methods:**

In a cross-sectional survey using the standardised protocol of the global programme, the child and adolescent versions of the HARDSHIP questionnaire, translated into the local languages, were completed by pupils in mediated sessions within their classes in nine schools representative of Zambia’s urban/rural divide. Headache diagnostic questions were based on ICHD-3 except for UdH, which we redefined as *mild or moderate* headache lasting < 1 h. Enquiry included multiple aspects of attributed burden.

**Results:**

Of 2,759 potential participants, 2,089 (615 children [29.4%], 1,474 adolescents [70.6%]) completed questionnaires (participating proportion 75.7%). The unfeasibly high observed prevalence of migraine reported earlier (51.6%, including probable migraine) was reduced by redefining UdH to 42.9%, still highly questionable. Nonetheless, burden measures clearly distinguished migraine from tension-type headache (TTH) and UdH. Migraine headache was the most intense, most frequent and longest lasting, but proportion of time in ictal state (pTIS) was only 1.3%. However, pTIS was much greater among those with probable medication-overuse headache (7.9%) or other headache on ≥ 15 days/month (9.0%). Headache accounted for an estimated loss of 3.5% of school time in those affected, migraine having twice the impact of TTH or UdH, while H15 + accounted for > 15% of lost school time. Almost one in six parents (15.9%) lost time from their own work. Migraine had greater impact than TTH and UdH on both emotional impact and quality-of-life scores.

**Conclusions:**

Headache, prevalent among children and adolescents in Zambia, is associated with burdens that include lost school time, with, potentially, lifelong negative impact. These findings are of importance to national educational and health policies. Our modified definition of UdH was a step towards diagnostic veracity, but did not resolve the diagnostic difficulties among young people. Future studies might consider further modifications, but full review of the ICHD criteria for migraine in these age groups is needed.

## Introduction

Following our initial report of headache prevalence among children (6–11 years) and adolescents (12–17) in Zambia [1], here we report revised estimates along with estimates of headache-attributed burden.

The reason for the revised prevalence estimates lies with diagnostic uncertainties among these age groups. In a study in Turkey [2], where the methodology was developed for the global schools-based programme of enquiry [2] conducted by the Global Campaign against Headache [3, 4], many participating pupils reported mild headache of short duration (< 1 h) [5], which could not be diagnosed by International Classification of Headache Disorders (ICHD) criteria [6]. A literature review showed that previously published studies of headache prevalence among children and adolescents had failed to account for large numbers of participants: that is, numbers with any headache were very much larger than the sums of those diagnosed with migraine or tension-type headache (TTH), usually with no indication of what headache type(s) might explain these differences [5]. We proposed that these headaches were expressions by the immature brain of what would later develop into migraine or TTH, and coined the term “undifferentiated headache” (UdH) [5].

Subsequent studies within the Global Campaign, in Austria [7], Lithuania [8], Georgia [9], Iran [10], Nepal [11] and Mongolia [12], gave support to this proposal, with migraine, TTH and UdH (as defined) all being prevalent. Burden estimates showed clear gradients (migraine > TTH > UdH), indicating that, at individual level, UdH was clearly differentiated from the others in burden measures, and the least troublesome of the three.

In studies in sub-Saharan Africa (SSA), however, we encountered difficulties: age- and gender-adjusted 1-year prevalence estimates for migraine (definite + probable) were unfeasibly high (in Ethiopia 38.6% [13], in Benin 53.4% [14], and here in Zambia 53.2% [1]). Interrogating the data, we became aware that diagnoses were very often dependent solely – and, we believed, very unreliably – on the highly subjective difference between “mild” and “moderate” headache [9, 14]. Arguing that short duration was the crucial feature of UdH, we proposed a modification of its criteria to *mild or moderate headache*, still of duration < 1 h [9].

Without biomarkers, the truth is undiscernible. Additionally, UdH cannot be defined biologically: it is a developing disorder (or disorders), therefore likely to be highly variable in presentation, with features of migraine or TTH. It is important, nonetheless, to recognise UdH epidemiologically, lest a large part of headache-attributed burden remain unaccounted for and its consequences unacknowledged. Here we present the revised prevalence estimates for migraine, TTH and UdH based on this modified definition of UdH, along with estimates of burden associated with these and with headache disorders characterised by headache on ≥ 15 days/month (H15+).

The purposes of this study were therefore three-fold. By applying the modified criteria for UdH we aimed to ameliorate, if not resolve, the uncertainties surrounding headache diagnosis among children and adolescents. We sought to inform national health and education policies. As in other studies in the global programme, we aimed to contribute to the Global Burden of Disease (GBD) study, and to add to knowledge and understanding of headache disorders among these age groups.

## Methods

This was a schools-based cross-sectional survey. Other than with regard to the definition of UdH, the detailed methodology has been published previously [1, 2]. The study was conducted during 2017 and 2018, being interrupted in late 2017 by a national cholera outbreak [15]. Collaboration was later disrupted by the SARS-CoV-2 pandemic, delaying analyses.

### Ethics and approvals

The research was conducted in accordance with the Declaration of Helsinki.

Approvals were obtained from the Excellence in Research and Science Converge Institutional Review Board (ERES IRB), from the Ministries of Health and Education, and from each school’s parent-teachers’ association, along with agreements of headteachers and class teachers.

Prior written parental consent for each participant was required by the terms of ethics approval. Information sheets describing the survey, along with consent forms, were distributed to pupils to take home to parents or guardians. Verbal assent was also required from each participating child or adolescent.

Data were collected anonymously, and data protection legislation complied with.

### Study procedures

We used the standardised protocol for the global series [2]. We purposively selected nine schools in Lusaka (urban) and Copperbelt (semi-rural), these two of Zambia’s ten provinces representing the country’s urban/rural divide [1]. The child and adolescent versions of the structured Headache-Attributed Restriction, Disability, Social Handicap and Impaired Participation (HARDSHIP) questionnaire, translated into Bemba and Nyanja (the local languages) following the Global Campaign’s translation protocol for lay documents [16], were completed by pupils in mediated sessions within their classes.

Demographic enquiry preceded headache diagnostic questions based on ICHD-3 [6], except for UdH. Burden enquiry was into multiple aspects (symptom burden, medication use, headache-attributed lost health, impaired participation [lost time from school and other activities], lost parental worktime, emotional impact and quality of life [QoL]), with timeframes of 4 weeks and 1 day (the latter based on headache yesterday [HY], when this occurred).

We performed independent double data-entry, with reconciliation by reference to the source data [1].

### Analysis

Initial analyses, with the conventional definition of UdH, were performed at University of Mersin, Turkey. Subsequent analyses with the modified definition were performed at Norwegian University of Science and Technology.

Diagnoses were made during analysis, following the HARDSHIP algorithm [17], first identifying H15+ (classified as probable medication-overuse headache [pMOH] when medication use on ≥ 14 of the preceding 28 days was also reported or, otherwise, as “other H15+”), then UdH (modified criteria), definite migraine, definite TTH, probable migraine and probable TTH in this hierarchical order [1]. In the analyses, we combined definite and probable migraine and definite and probable TTH.

We recorded frequencies of headache and of acute medication intake in days/4 weeks. Headache intensity was reported as “mild”, “moderate” or “severe”, which we converted to a numerical scale of 1–3, treating this as continuous. Duration of episodes was reported in hours as < 1, 1–2, 2–4 or > 4, which we converted for analysis by taking the mid-points (0.5, 1.5, 3 and 8 respectively, assuming a range of 4–12 h for the last). We calculated proportion (%) of time in ictal state (pTIS) from frequency and duration. We estimated lost health at individual level (%) as pTIS*DW, where DW, on a scale 0–1, was the disability weight attributed in the GBD study to the ictal state of the headache type (0.441 for migraine, 0.037 for TTH, 0.223 for pMOH; 0 indicating no health loss and 1 a health loss equivalent to being dead [18]). We estimated lost health at population level by factoring in prevalence.

We made prevalence estimates as proportions (%), adjusting observed values for age and gender using population statistics for Zambia (in 2018) from The World Bank [19].

We counted days of lost school time in the preceding 4 weeks, reckoning not going to school as a whole day lost and leaving school early as a half-day lost, and calculated proportions using a denominator of 20. We separately counted days of limited activity (defined as “could not do things you wanted to because of your headaches”), with, for proportions, a denominator of 28. We calculated proportions reporting a lost school day yesterday because of HY, and predicted values of lost school yesterday from the product of number affected by headache and mean reported days (divided by 20) lost per pupil over the preceding 4 weeks. We counted parents of participating pupils who had, according to the pupils, lost time from their own work (regardless of quantity), also over the preceding 4 weeks. Pupils’ responses to questions addressing concentration, emotional impact and QoL were all on a 4-point Likert scale (“never”, “sometimes”, “often”, “always”) [2], again referring to the preceding 4 weeks. We scored these 0–3, and summed them to generate an emotional impact score (including concentration; potential range 0–18, high being adverse) and a QoL score (0–36, low being adverse) [2].

In descriptive statistics we used means, standard deviations (SDs) and standard errors (SEs) for continuous variables, and proportions (%) with 95% confidence intervals (CIs) for categorical data. We used Kruskal-Wallis with post hoc Dunn’s test to evaluate group differences in continuous variables, and binary logistic regression to evaluate group differences in binary outcomes.

Population-based estimates of burden were derived from our revised prevalence estimates, with UdH redefined as mild or moderate headache lasting < 1 h.

In all analyses, we set significance at *p* < 0.05.

## Results

### Sample description

Of 2,759 potential participants (eligible pupils present on the day), 2,089 completed the questionnaires (children 615 [29.4%], adolescents 1,474 [70.6%]; male 1,128 [54.0%], female 961 [46.0%]) [1]. Children and, less so, females were therefore underrepresented, for which statistical corrections were necessary. The participating proportion was 75.7% (2,089/2,759) [1].

### Diagnostic enquiry

Table 1 shows the proportions of participants responding positively to each of the diagnostic questions, categorised according to the diagnosis made, with modified criteria for UdH applied.

The differences between migraine and TTH (each including probable diagnoses) were largely self-fulfilling, given that, in ICHD, TTH criteria are essentially the negation of migraine criteria [6]. For UdH, duration and intensity were driven by its diagnostic criteria, mandating duration of < 1 h and precluding severe headache. Otherwise, UdH was mostly mid-placed, although unilaterality, reported by fewer than one third (30.3%) of participants with migraine, and by only 19.6% with TTH, was most common (35.5%) among those with UdH (Table 1). Pulsating headache was reported by over two thirds with migraine (69.7%), rather fewer (60.5%) with UdH, but by only one half (49.6%) with TTH. Aggravation of headache by physical activity and causing avoidance of exercise were reported more by those with migraine (72.6%; 64.0%) than by those with UdH (46.5%; 49.5%) or TTH (42.9%; 41.5%).

Nevertheless, diagnostic distinction was attributable much more to presence or absence of associated symptoms than to headache characteristics, with UdH again mid-placed (Table 1). It is noteworthy, however, that phonophobia (but not photophobia) was reported by 85–90% of participants with headache, regardless of diagnosis.

**Table 1:** Responses to diagnostic questions by diagnosis made

	Migraine^1^ (*N* = 897)	Tension-type headache^1^ (*N* = 224)	Undifferentiated headache (*N* = 602)
	*n* (%)		
**Headache characteristics**			
duration: < 1 h 1–2 h > 2 h missing	88 (10.0) 381 (43.3) 411 (46.7) 17	9 (4.2) 155 (71.8) 52 (24.2) 8	602 (100) 0 0 0
intensity: mild moderate severe missing	94 (10.6) 414 (46.6) 380 (42.8) 9	61 (27.7) 121 (55.0) 38 (17.3) 4	312 (51.8) 290 (48.2) 0 0
unilateral	272 (30.3%)	44 (19.6%)	214 (35.5%)
pulsating	625 (69.7)	111 (49.6%)	364 (60.5%)
aggravated by physical activity	651 (72.6%)	96 (42.9%)	280 (46.5%)
causing avoidance of exercise	574 (64.0%)	93 (41.5%)	298 (49.5%)
**Associated symptoms**			
nausea	539 (60.1%)	20 (8.9%)	200 (33.2%)
vomiting	347 (38.7%)	10 (4.5%)	103 (17.1%)
photophobia	561 (62.5%)	31 (13.8%)	223 (37.0%)
phonophobia	814 (90.7%)	191 (85.3%)	511 (84.9%)
photophobia + phonophobia	531 (59.2%)	22 (9.8%)	198 (32.9)

^1^Including definite and probable diagnoses

### Revised prevalence estimates

Observed 1-year prevalence of any headache was 87.3% (*n* = 1,824), slightly higher among females (89.8%) than males (85.2%; *p* < 0.01), and higher among adolescents (89.8%) than children (81.5%; *p* < 0.01). HY was reported by 22.2% of the sample. These findings have been reported previously [1].

Table 2 shows observed prevalence of each headache type under the modified criteria for UdH. For comparison, the table also shows the previously reported values (with conventional criteria for UdH) for migraine, TTH and UdH [1] (those for pMOH [1.1%] and other H15+ [2.9%] being unaffected). Observed 1-year prevalence of UdH increased from 14.8% to 28.8%. However, migraine (reduced from 51.6% to 42.9%) remained by far the most common headache type. TTH was considerably less prevalent (reduced from 14.7% to 10.7%).

**Table 2:** Observed 1-year prevalence of headache overall and of each headache type according to modified and conventional criteria for undifferentiated headache

Headache type	Modified criteria^1^	Conventional criteria^2^ (from [1])
	% [95% confidence interval]	
Any headache^3^	87.3 [85.9–88.7]	87.3 [85.9–88.7]
UdH	28.8 [26.9–30.8]	14.8 [13.3–16.3]
Migraine	42.9 [40.8–45.1]	51.6 [49.5–53.7]
TTH	10.7 [9.4–12.1]	14.7 [13.2–16.2]
pMOH^3^	1.1 [0.7–1.5]	1.1 [0.7–1.5]
Other H15 + ^3^	2.9 [2.2–3.6]	2.9 [2.2–3.6]
Unclassified	0.8	2.3

UdH: undifferentiated headache; TTH: tension-type headache; pMOH: probable medication overuse headache; H15+: headache on ≥15 days/month; ^1^mild or moderate headache lasting <1 hour; ^2^mild headache lasting <1 hour; ^3^these were unaffected by the definition of UdH

Table 3 shows observed prevalence of each headache type by age and gender, along with values adjusted for these variables (the latter differing very slightly from those previously published [1] because here we used smaller [1-year] age-bins in the adjustments). Significant differences between genders (females > males) were seen only for all headache, and between age groups (adolescents > children) only for all headache and UdH.

### Burden

Table 4 shows the symptom burden associated with each headache type. Overall headache frequency was 2.9 days/4 weeks, with very skewed distribution because of H15+. Mean overall headache duration was 2.3 h, with intensity of 2.0 (moderate). Of the episodic headaches, migraine was the most frequent, longest lasting (therefore associated with substantially the highest pTIS) and most intense. Those with H15+ (pMOH or other) had headache on about two thirds of all days, but lasting on average for only 2.3–2.5 h, so that mean pTIS did not exceed 9%. Both pMOH and other H15 + were reportedly as intense as migraine (moderate to severe) (Table 4).

Table 5 shows frequency of symptomatic medication use, along with frequency of headache (from Table 4) for comparison. These data reveal that, except for pMOH, not all headache days were medicated. This is very apparent for other H15+.

**Table 3:** Observed 1-year prevalence by headache type, gender and age according to modified criteria for undifferentiated headache, and age- and gender-adjusted prevalence estimates

	All headache (*n* = 1,824)	Migraine (*n* = 897)	TTH (*n* = 224)	pMOH (*n* = 23)	Other H15+ (*n* = 61)	UdH (*n* = 602)
**Observed prevalences** (% [95% confidence interval])						
Overall	87.3 [85.9–88.7]	42.9 [40.8–45.1]	10.7 [9.4–12.1]	1.1 [0.7–1.5]	2.9 [2.2–3.6]	28.8 [26.9–30.8]
Gender male (*n* = 1,128) female (*n* = 961)	**85.2** [83.1–87.2] **89.8** [87.9–91.7]	41.2 [38.3–44.1] 45.0 [41.8–48.1]	11.3 [9.4–13.1] 10.1 [8.2–12.0]	1.2 [0.5–1.8] 1.0 [0.4–1.7]	2.7 [1.8–3.7] 3.1 [2.0-4.2]	28.1 [25.5–30.7] 29.7 [26.8–32.5]
Age group (years) 6–11 (*n* = 615) 12–17 (*n* = 1,474)	**81.5** [78.4–84.6] **89.8** [88.3–91.4]	42.4 [38.5–46.3] 43.1 [40.6–45.7]	9.9 [7.6–12.3] 11.1 [9.5–12.7]	0.8 [0.1–1.5] 1.2 [0.7–1.8]	2.0 [0.9-3.0] 3.3 [2.4–4.2]	**25.5** [22.1–29.0] **30.2** [27.8–32.5]
**Age- and gender-adjusted prevalence estimates** (% [95% confidence interval])						
Overall	85.7 [84.1–87.2]	42.9 [40.7–45.0]	9.1 [7.9–10.5]	0.9 [0.5–1.4]	2.4 [1.8–3.2]	29.7 [27.7–31.7]

TTH: tension-type headache; pMOH: probable medication-overuse headache; H15+: headache on ≥ 15 days/month; UdH: undifferentiated headache; significantly different values (*p* < 0.05) are emboldened

**Table 4:** Symptom burden by headache type and overall

Headache type	Headache frequency days/4 weeks (mean±SD)	Headache duration hours (mean±SD)	Estimated pTIS % (mean±SE)	Headache intensity scale 1–3^1^ (mean±SD)
**Episodic headaches**				
Migraine	2.5±2.7	3.5±2.9	1.3±0.1	2.3±0.7
Tension-type headache	1.9±2.4	2.5±2.2	0.6±0.1	1.9±0.7
Undifferentiated headache	1.7±2.4	0.5± < 0.1	0.1± < 0.1	1.5±0.5
**Headache on ≥ 15 days/month**				
Probable medication-overuse headache	20.7±4.7	2.7±3.1	7.9±1.9	2.5±0.7
Other headache on ≥ 15 days/month	19.4±5.2	3.0±3.0	9.0±1.3	2.3±0.6
**All headache** ^**2**^	2.9±4.6	2.3±2.6	1.2±0.1	2.0±0.7

SD: standard deviation; SE: standard error; pTIS: proportion of time in ictal state; ^1^equating 1-3 to the reported categories “mild”, “moderate” and “severe”, and treating as continuous data; ^2^includes unclassified headache

**Table 5:** Symptomatic medication use by headache type and overall

Headache type	Pupils responding *n* ^1^	Medication use days in preceding 4 weeks (mean ± SD)	Headache frequency days/4 weeks (mean ± SD) (from Table 4)
**Episodic headaches**			
Migraine^1^	865	1.6±2.2	2.5±2.7
Tension-type headache	220	1.0±1.7	1.9±2.4
Undifferentiated headache	590	0.9±1.6	1.7±2.4
**Headache on ≥ 15 days/month**			
Probable medication-overuse headache	23	20.1±4.6	20.7±4.7
Other headache on ≥ 15 days/month	58	3.0±3.8	19.4±5.2
**All headache** ^**1,2**^	1,771	1.6±3.0	2.9±4.6

SD: standard deviation; ^1^values reflect that not all pupils responded to this enquiry; ^2^includes unclassified headache

**Table 6:** Headache-attributed lost health by headache type

Headache type	Disability weight (from [18])	Age- and gender-adjusted prevalence % (from Table 3)	Proportion of time in ictal state (pTIS) %		Lost health %	
			Individual (from Table 4)	Population	Individual^1^ (mean)	Population^2^
Migraine	0.441	42.9	1.3	0.6	0.6	0.2
Tension-type headache	0.037	9.1	0.6	0.1	0.02	0.002
Probable medication-overuse headache	0.223	0.9	7.9	0.1	1.8	0.02

^1^Calculated as the product of pTIS and disability weight; ^2^calculated as the product of mean individual lost health and adjusted prevalence (with apparent discrepancies due to rounding)

Table 6 shows the estimated health loss associated with each headache type, at individual and population levels. The percentages indicate corresponding reductions in healthy life expectancy. Since these estimates depended on the DWs allocated in GBD to migraine, TTH and pMOH [18], only these types were included. At individual level, a substantial health loss of 1.8% was attributed to pMOH. A lesser individual health loss of 0.6% was attributed to migraine, but, with its much higher prevalence, migraine accounted for a 10-fold higher loss at population level (and 100-fold compared with TTH) (Table 6).

**Table 7:** Lost time from school and other activities because of headache in the preceding 4 weeks, by headache type and overall, and by age group

Headache type	Headache frequency days/4 weeks (mean)	Days of lost school	Days of limited activity
		days/4 weeks/affected pupil (mean ± SD)	
**Episodic headaches**			
Migraine children adolescents	2.5 2.2 2.6	0.7±1.5 0.6±1.4 0.7±1.5	1.5±2.2 1.3±2.1 1.5±2.2
Tension-type headache children adolescents	1.9 1.7 1.9	0.4±1.0 0.3±0.6 0.4±1.1	0.8±1.5 0.6±1.1 0.9±1.6
Undifferentiated headache children adolescents	1.7 1.8 1.7	0.4±1.3 0.4±1.4 0.4±1.2	0.7±1.5 0.7±1.6 0.7±1.5
**Headache on ≥ 15 days/month**			
Probable medication-overuse headache children adolescents	20.7 24.2 19.7	3.0±4.8 2.6±5.8 3.1±4.6	8.5±8.7 14.0±10.3 6.9±7.9
Other headache on ≥ 15 days/month children adolescents	19.4 18.8 19.5	3.6±5.9 7.1±7.8 2.8±5.2	7.2±7.8 8.3±8.9 6.9±7.6
**All headache** ^**1**^ children adolescents	2.9 2.6 3.1	0.7±1.9 0.7±2.1 0.7±1.8	1.4±2.9 1.3±2.9 1.4±2.9

SD: standard deviation; ^1^includes unclassified headache

Table 7 shows impaired participation (lost time from school and other activities, including playtime) because of headache overall and by headache type. In each age group, all headache accounted for 0.7 days of missed school each 4 weeks, a loss of 3.5% assuming a 5-day week. Days of limited activities were double this, although the denominator for these was presumed to be 28 days. Migraine had greater impact than TTH or UdH, but H15 + had far more serious impact: 3.0 days lost from school equates to 15% of school time, while 7.1 days (children with other H15+) equates to over one third (35%) of school time. Children with pMOH reported 14 days of limited activities (Table 7): one half of all days (although this was based on *n* = 5). Adolescents with pMOH (*n* = 18) were reportedly less impacted (one quarter of all days).

Table 8 presents estimates of lost school time derived from data related to HY. It includes predictions based on recalled lost days/4 weeks (in other words, it compares actual lost school days yesterday, an objective measure, with expected lost school days yesterday according to pupils’ recall of past lost days). The differences were not large: for all headache, the two estimates matched exactly. However, counts of actual losses, it should be noted, would have excluded lost days immediately preceding weekends or school holidays, since all data collection was carried out on school days.

**Table 8:** Lost school days yesterday because of headache yesterday (HY), by headache type and overall, with predictions based on recalled lost days/4-week

Diagnosed headache type	Pupils affected *n* (from Table 3)	Proportion reporting HY *n* (% of those with headache type) (from [1])	Missed school day yesterday *n* (% of those reporting HY)	
			Actual	Predicted
**Episodic headaches**				
Migraine	897	258 (28.8)	37 (14.3)	31 (12.0)
Tension-type headache	224	42 (18.8)	5 (11.9)	5 (11.9)
Undifferentiated headache	602	111 (18.4)	13 (11.7)	12 (10.8)
**Headache on ≥ 15 days/month**				
Probable medication-overuse headache	23	11 (47.8)	2 (18.2)	3 (27.3)
Other headache on ≥ 15 days/month	61	39 (63.9)	6 (15.4)	11 (28.2)
**All headache** ^**1**^	1,824	464 (25.4)	64 (13.8)	64 (13.8)

^1^Includes unclassified headache

Table 9 shows impact on parents: specifically, lost parental worktime in the preceding 4 weeks because of a son’s or daughter’s headache. Almost one in six parents (15.9%) lost at least some time (pupils were not expected to be aware of how much), those of children (21.0%) more than those of adolescents (14.0%), a pattern repeated for all headache types except pMOH (with low numbers). A quarter (25.7%) of parents of children with migraine lost time, but even higher proportions of parents of adolescents with pMOH and of parents of children or adolescents with other H15+ (Table 9).

**Table 9:** Parents’ lost work in the preceding 4 weeks, by pupils’ headache type and overall, and by age group

Headache type	Pupils affected *n* (from Table 3)	Headache frequency days/4 weeks (mean) (from Table 7)	Parent missed work *n* (% of pupils with headache type)
**Episodic headaches**			
Migraine children adolescents	897 261 636	2.5 2.2 2.6	176 (19.6) 67 (25.7) 109 (17.1)
Tension-type headache children adolescents	224 61 163	1.9 1.7 1.9	20 (8.9) 8 (13.1) 12 (7.4)
Undifferentiated headache children adolescents	602 157 445	1.7 1.8 1.7	60 (10.0) 22 (14.0) 38 (8.5)
**Headache on ≥ 15 days/month**			
Probable medication-overuse headache children adolescents	23 5 18	20.7 24.2 19.7	9 (39.1) 1 (20.0) 8 (44.4)
Other headache on ≥ 15 days/month children adolescents	61 12 49	19.4 18.8 19.5	21 (34.4) 6 (50.0) 15 (30.6)
**All headache** ^**1**^ children adolescents	1,824 501 1,323	2.9 2.6 3.1	290 (15.9) 105 (21.0) 185 (14.0)

^1^Includes unclassified headache

Table 10 compares the headache types for these impaired-participation measures (lost school time, lost time from other activities, and lost parental worktime). On all of these measures, TTH and UdH were clearly distinguished from migraine, but not from each other, while pMOH was clearly distinguished from the episodic headaches but not from other H15+.

Table 11 (which includes similar between-type comparisons) shows emotional impact and QoL scores, neither of which have intuitive meaning, although the latter can be compared with normative data from the participants with no headache. Emotional impact scores did not differ between UdH and TTH, but were significantly higher (more adverse) for migraine. Numerically, pMOH scored highest (most adverse), but not significantly against migraine. All headache types had significant impact on QoL, with a clear gradient (H15 + > migraine > TTH or UdH).

**Table 10:** Comparisons of headache types for measures of impaired participation

	*n* ^1^	mean±SD; SE	Between-type significance *p* (Kruskal-Wallis with post-hoc Dunn’s test)			
			TTH	UdH	pMOH	Other H15+
**Lost school days/4 weeks**						
Migraine	829	0.7±1.5; 0.1	**< 0.001**	**< 0.001**	**< 0.001**	**< 0.001**
TTH	219	0.4±1.0; 0.1		0.94	**< 0.001**	**< 0.001**
UdH	577	0.4±1.3; 0.1			**< 0.001**	**< 0.001**
pMOH	23	3.0±4.8; 1.0				0.55
Other H15+	60	3.6±5.9; 0.8				
**Lost days/4 weeks from other activities**						
Migraine	848	1.5±2.2; 0.1	**< 0.001**	**< 0.001**	**< 0.001**	**< 0.001**
TTH	221	0.8±1.5; 0.1		0.14	**< 0.001**	**< 0.001**
UdH	587	0.7±1.5; 0.1			**< 0.001**	**< 0.001**
pMOH	23	8.5±8.7; 1.8				0.89
Other H15+	59	7.2±7.8; 1.0				
**Lost parental worktime** n^1^ (%) of parents of pupils with headache type reported to have lost time in preceding 4 weeks						
Migraine	176	19.6%	**0.002**	**< 0.001**	**0.02**	0.06
TTH	20	8.9%		0.99	**0.001**	**< 0.001**
UdH	60	10.0%			**< 0.001**	**< 0.001**
pMOH	9	39.1%				0.99
Other H15+	21	34.4%				

TTH: tension-type headache; UdH: undifferentiated headache; pMOH: probable medication-overuse headache; H15+: headache on ≥ 15 days/month; ^1^values reflect that not all pupils responded to every enquiry; significant p-values are emboldened

**Table 11:** Emotional impact and quality of life scores by headache type

Headache type	Pupils responding *n* ^1^	Score mean ± SD	Between-type significance *p* (Kruskal-Wallis with post-hoc Dunn’s test)			
			TTH	UdH	pMOH	Other H15+
**Emotional impact** (0–18; higher scores adverse)						
Migraine	860	8.4±3.3	**< 0.001**	**< 0.001**	0.24	0.11
TTH	219	7.3±3.5		0.17	**0.01**	**< 0.001**
UdH	585	7.0±3.4			**0.002**	**< 0.001**
pMOH	23	9.3±3.4				0.89
Other H15+	59	9.0±3.2				
All headache^2^	1,759	7.8±3.4				
**Quality of life** (0–36; lower scores adverse)						
No headache	253	23.6±5.6	*p* < 0.001 against all headache types			
Migraine	848	19.6±6.1	**0.003**	**< 0.001**	0.12	**< 0.001**
TTH	209	21.1±6.3		0.23	**0.01**	**< 0.001**
UdH	565	21.8±5.9			**< 0.001**	**< 0.001**
pMOH	23	17.8±5.2				0.55
Other H15+	55	16.7±6.7				
All headache^2^	1,824	20.4±6.2				

TTH: tension-type headache; UdH: undifferentiated headache; pMOH: probable medication-overuse headache; headache on ≥15 days/month; ^1^values reflect that not all pupils responded to every enquiry; ^2^includes unclassified headache; significant p-values are emboldened

## Discussion

We do not comment on the prevalence estimates except with regard to their dependence on the definition of UdH. As already noted [1], headache disorders are common among children and adolescents in Zambia (age- and gender-adjusted 1-year prevalence estimate: 85.7%), much as they are in Ethiopia (72.8% [13]) and Benin (88.6% [14]), two other SSA countries. Also as in these countries, according to symptoms reported, migraine is the most common type.

The key issue addressed here – and, clearly, not fully resolved – was the inherent imprecision in epidemiological diagnosis of headache type in children and adolescents. Partly this arises from the difficulties of enquiry among these age groups (mitigated here by mediated enquiry), and the unavoidable dependence on largely subjective responses; but partly, and perhaps more, it is engendered by the ICHD diagnostic criteria themselves, which do not apply well to these age groups [20]. As we have previously noted, in these age groups, with headache disorders evolving from an undifferentiated form as the brain matures [5], diagnostic ambiguities appear inevitable [9–14].

The unfeasibly high observed prevalence of migraine reported earlier (51.6% [1]) was reduced here, by redefining UdH, to 42.9%, the drop entirely accounted for by reduction of probable migraine. While this somewhat lower estimate remains highly questionable, it should be noted that burden estimates clearly distinguished headache classified as migraine from headache classified as TTH or UdH, as we shall show. We discuss these estimates now.

The not inconsiderable symptom burden of headache among these young people was expressed in headache of moderate mean intensity (2.0 on a 1–3 scale) and an overall frequency of 2.9 days/4 weeks, albeit with short mean duration (2.3 h). Of the episodic headaches, migraine was the most intense, most frequent and longest lasting, but pTIS for migraine was, nonetheless, only 1.3%. Probably reflecting the short durations, only about half of headache days were medicated among those with episodic headache, regardless of diagnosis. Participants with H15+, moderate to severe in intensity, reported headache on about two thirds of all days, still short-lasting (mean 2.3–2.5 h) but with pTIS of 7.9-9.0% - a large and meaningful proportion of their time. Notably, while those with pMOH used acute medication on 20 days (as many as they had headache), those with other H15 + did not (3 *versus* 19 days), suggesting low risk of developing pMOH. Health loss estimates depended entirely on pTIS, which was multiplied by DW [18], a constant for each headache type. At individual level, as expected, estimated health loss from migraine (0.6%) greatly exceeded that from TTH (0.02%), but was itself much exceeded by the health loss from pMOH (1.8%). No DW exists for UdH.

What is always of concern in young people is impaired participation: lost time from day-to-day activities, importantly including schooling, although the value of play and social activities should not be discounted. All headache, among those affected, was responsible for an estimated loss of 3.5% of all school time. Migraine had about double the impact of TTH or UdH, but H15 + was responsible for a very much more worrying 15% lost school time, while children with other H15 + reportedly lost a seriously concerning 35% of school time. These estimates were dependent on pupils’ recall, while those based on what happened yesterday with HY were objective (although based on smaller numbers). For all headache, the two estimates matched exactly. However, since the survey was always performed on school days, counts of actual lost days yesterday would not have included any lost days immediately preceding weekends or school holidays (at least 20%).

Impact on parents, and on their work, is an often-unrecognised element of headache-attributed burden. Almost one in six parents (15.9%) lost at least some time, those of adolescents (14.0%) less than those of children (21.0%), probably reflecting waning dependence.

Because the emotional impact and QoL scores lack intuitive meaning, they are difficult to interpret. Neither showed much difference in impact between TTH and UdH, but, on each score, migraine had significantly greater impact than both, while H15 + had numerically but not significantly greater impact than migraine.

Our principal purpose in Table 10, comparing the headache types for the measures of impaired participation, was to discover whether the diagnostic categories, including UdH according to the modified definition, were distinguished by these measures from each other. Lost school time, lost time from other activities and lost parental worktime all clearly distinguished migraine from TTH and UdH, but not TTH from UdH. In Table 11, emotional impact and QoL scores also clearly distinguished migraine from TTH and UdH. These findings suggest, quite strongly we believe, that pupils diagnosed with migraine were reporting a headache measurably and consistently more burdensome than those diagnosed with TTH or UdH. In other words, the diagnostic categorisation had some demonstrable coherence, notwithstanding the very high proportion diagnosed with migraine.

Table 1, showing responses to the diagnostic questions according to diagnosis made (migraine, TTH or UdH), offers additional insight. As noted, diagnostic distinction was attributable more to presence or absence of associated symptoms than to headache characteristics. With regard to the latter, migraine and UdH were most clearly distinguished from each other by aggravation by physical activity and avoidance of exercise, each of these likely to impair participation. Among associated symptoms, phonophobia had little diagnostic value, being reported by 85–90% of participants with headache regardless of diagnosis. This has been reported before: we saw similar proportions in Benin (87.8% [14]) and Ethiopia (78.9% [13]). Nausea and photophobia (both expected to contribute to disability) were notably different between diagnoses (migraine 60–63%, UdH 33–37%, TTH 9–14%).

But, as we said earlier, without biomarkers, and since UdH cannot be defined biologically, the truth is undiscernible. Our modified definition of UdH does not appear to be more than a step towards diagnostic veracity. Further modifications might be fruitful: we leave this to future studies, but a full review of the ICHD criteria for migraine in childhood and adolescence is also very obviously called for. Epidemiologically, and for purposes of health and educational policies, what matters is to recognise all headache-attributed burden and its consequences. Diagnoses that inform treatment need to be correct, but epidemiologically we observe a broad spectrum of headache, of which only the very tip of one end is seen in clinic. This point cannot be overemphasised.

There are no published studies, using the modified criteria for UdH, available for comparison.

Our findings add to our general knowledge and understanding of headache among these age groups. They can also inform health and educational policies in Zambia. With regard to health policy, the evidence of impaired health and wellbeing due to headache calls for a formal needs assessment for paediatric headache care. It also calls for health education, perhaps focusing on appropriate medication use. By our estimates, only 0.9% of pupils in Zambia have pMOH, but a further 2.4% with other H15 + are at risk of it. Our adult study in Zambia, performed in the same regions, found a much higher pMOH prevalence of 7.1% [21], which early health education might do much to avert. Educational policy in Zambia has to deal with high levels of absenteeism, especially in rural areas [22]. Poorly educated parents see little value in education, and no need for their children to attend classes. Traditional cultural practices such as puberty rites may enforce seclusion of pubertal girls for a period of time [22]. Many children abscond from classes without their parents’ knowledge, through boredom, dislike of teachers, bullying, laziness and moodiness, or to impress friends [22]. Lost schooling because of headache (on average, 0.7 days of each 4 weeks [3.5%], and much more among those with H15+) is apparently on top of all these other causes.

### Strengths and limitations

Strengths of this study lay in the tested and validated methodology [2] and adequate sample size [23]. The comparisons between estimates dependent on pupils’ recall and those based on HY, part of the standardised methodology, with the two matching exactly for all headache, was a particular strength. The limitations relating to the diagnostic uncertainties have, we believe, been sufficiently discussed. Others were identified previously [1]. The participating proportion (75.7%) was good but not optimal, reduced by the requirement for prior written parental consent rather than informed non-objection. The sampling process failed to equalise numbers across the age groups, and schools-based sampling in Zambia has inbuilt bias against female adolescents [24]. These factors had a limiting effect on the representativeness of the sample, for which statistical correction provided only a partial solution. Logistic and practical difficulties included high numbers per class in some schools, poor literacy requiring questions to be read and explained to many children, and the cholera outbreak mid-study [15].

## Conclusions

Headache is prevalent among children and adolescents in Zambia and, whatever the diagnostic divides, it is associated with burdens that include lost school time, with, potentially, lifelong negative impact. These findings add to our knowledge and understanding of headache in these age groups. They also inform health and educational policies in Zambia.

The broader issue of diagnosing headache correctly in these age groups – or even defining the types of headache that children and adolescents experience – is not fully resolved. Our modified definition of UdH was only a step towards diagnostic veracity, and future studies might consider further modifications, perhaps informed by interrogation of large datasets [25]. In any case, we are not the first to question the ICHD criteria for migraine in these age groups [20], and a full review is quite obviously needed. The unquestionable importance of correct diagnosis in clinical practice is not matched in epidemiological enquiry for purposes of public health and health policy, but it remains an issue of more than academic interest.

## Data Availability

The data are held on file at University of Mersin and the analytical subset also at Norwegian University of Science and Technology. Once analysis and publications are completed, they will be freely available for non-commercial purposes to any person requesting access in accordance with the general policy of the Global Campaign against Headache.

## Abbreviations

CI: Confidence interval
DW: Disability weight
GBD: Global Burden of Disease (study)
H15+: Headache on ≥ 15 days/month
HARDSHIP: Headache-Attributed Restriction, Disability, Social Handicap and Impaired Participation (questionnaire)
HY: Headache yesterday
ICHD: International Classification of Headache Disorders
LTB: Lifting The Burden
MOH: Medication-overuse headache
pMOH: Probable MOH
pTIS: Proportion of time in ictal state
QoL: Quality of life
SD: Standard deviation
SE: Standard error (of the mean)
SSA: Sub-Saharan Africa
TTH: Tension-type headache
UdH: Undifferentiated headache
